# Ultra-Orthodox Nursing Students’ Cultural Challenges Inside and Outside Their Community during the COVID-19 Pandemic

**DOI:** 10.3390/ijerph19159215

**Published:** 2022-07-28

**Authors:** Sara Genut, Yaacov G. Bachner, Zvika Orr, Adi Finkelstein

**Affiliations:** 1 Department of Bioinformatics, Faculty of Life and Health Sciences, Jerusalem College of Technology, 7 Beit Hadfus Street, Jerusalem 95483, Israel; 2 Department of Epidemiology Biostatistics and Community Health Sciences, Ben-Gurion University of the Negev, Beer Sheva 84105, Israel; bachner@bgu.ac.il; 3 Department of Nursing, Faculty of Life and Health Sciences, Jerusalem College of Technology, 7 Beit Hadfus Street, Jerusalem 95483, Israel; orr@g.jct.ac.il (Z.O.); afinkels@g.jct.ac.il (A.F.)

**Keywords:** ultra-Orthodox, transcultural, minority, cultural competence, COVID-19, mixed methods, nursing students

## Abstract

In line with findings that nurses from minority groups have an important role in making health services accessible to their community, our study aimed to identify the challenges ultra-Orthodox Jewish nurses faced during COVID-19 in their encounters with patients and health staff from other communities, as well as their own community. The ultra-Orthodox community is a highly religious group that maintains isolation from general society, a phenomenon that affected its member experiences during COVID-19. Our research followed sequential explanatory mixed methods. The quantitative phase included a questionnaire completed by 235 female students (111 ultra-Orthodox and 124 non-ultra-Orthodox), followed by a qualitative phase, which included six focus-groups (*n* = 15). The quantitative analysis showed that the ultra-Orthodox students felt a higher sense of responsibility toward their community. They used their authority and knowledge to guide their community during the pandemic. The qualitative analysis identified two themes expressed as challenges ultra-Orthodox nursing students encountered within their community and with other sections of Israeli society. Our research shows the important role that transcultural nurses play in mediating updated health information otherwise inaccessible to their community, especially in times of crises. It is important to address dilemmas this group faces inside and outside their respective communities.

## 1. Introduction

Nurses must recognize patients’ needs based on their cultural background [1]. The recommendations of culturally competent baccalaureate nurses to be achieved during nursing education include awareness of personal culture, values, beliefs, attributes, and behaviors; skills in communicating with individuals from other cultures; and cultural knowledge and opportunities for students to interact with different cultures [2,3,4].

Providing culturally competent healthcare to diverse populations is important for reducing health inequalities and loss of critical information [5,6] and increasing patient satisfaction, which is a key factor in quality of care and correlated with patient compliance [7]. Studies have shown that language barriers [8] and lack of trust in the healthcare system [9] were reasons why minority populations were dissatisfied with the healthcare services they received [10]. Instead, they were likely to be satisfied with the healthcare services received from providers with the same race or ethnicity [11,12].

Considerable research has been performed on transcultural nurses as essential frontline workers providing culturally appropriate services to minority groups [13,14,15,16]. Transcultural nursing and ethnic minority nurses are attracting attention because of their central role in making medical care accessible to patients from diverse cultural backgrounds, and especially to patients in their community [14,15,17,18,19].

One of the goals of the US National Coalition of Ethnic Minority Nurse Associations, specifically highlighted during the COVID-19 pandemic, includes increasing the number of ethnic minority nurses because of their special contribution to society [20]. Dawson (2021) [17] described AA/Black nurses as frontline healthcare providers to patients in hospitals and nursing homes during this pandemic. Given their large network, they were among the first to inform and educate the AA/Black communities regarding COVID-19, working with politicians, social service agencies, healthcare systems, public health departments, etc.

## 2. The Aim of the Study

Our study aim was to examine the challenges of female Jewish ultra-Orthodox (UO) nursing students in encounters with other communities and their roles within their own community, especially during the COVID-19 pandemic.

Unlike other minorities, the ultra-Orthodox community stays isolated from the general society as part of religious ideology to preserve its traditional beliefs and behavior, a phenomenon that has both social and economic implications [21,22]. These implications influenced the experiences of the community during the COVID-19 pandemic. Ultra-Orthodox people made COVID-19-related decisions based on both health and medical rationalizations and religious considerations, and among them, obedience to their community leaders [23]. This community possesses characteristics typical of minority groups, which make it difficult to cope with the COVID-19 pandemic, such as large families, low socioeconomic status, and crowded living conditions [24]. This was part of the reason why a disproportionately large number of UO people contracted this disease and died [25].

## 3. Background

### 3.1. The Characteristic of the Ultra-Orthodox Community in Israel

According to the [26] (p. 40), in the general Jewish Israeli population of 2016, 14% defined themselves as UO, 16% as religious or very religious, 25% as traditional, and 45% as secular.

Important factors to consider in relation to religion and health are perceptions, behaviors, and access to health-related information [27]. First, the UO community lives an intense social and community life, built around prayers in synagogues, studying in groups, and multi-participant social events [24,28,29,30]. To keep themselves isolated from the influence of the so-called secular culture, they object to the use of virtual media and do not use TV or smartphones [31,32]. This factor was crucial in coping with COVID-19 as they lacked access to updated information about the virus and about measures taken by authorities, such as physical distancing. Due to their ideological non-use of the Internet, they were unable to use services remotely, such as food shopping, work, healthcare, and distance learning [33].

Second, there is a science literacy gap between UO and secular education, as sciences are either not taught or taught at a minimal level in UO schools. Moreover, relevant scientific knowledge is accessible nowadays in the media, yet, due to their ideological desire to remain isolated from the secular society, their members forsake the Internet, thereby lacking access to updated scientific knowledge and information [23,34].

Finally, the UO community mistrusts state authorities, which are perceived as interfering with their social isolation [31]. Thus, official instructions regarding COVID-19 were taken by this community with suspicion. Ultra-Orthodox leaders significantly shape believers’ perceptions in ways that affect health promotion and use of health services [23]. They influence the health behaviors as well as health-related decisions of individuals, often using “health mediators” between the UO community and professionals and service providers outside the UO world [35].

### 3.2. Ultra-Orthodox Jewish Nurses in Israel

Ultra-Orthodox Jewish nursing students represent a new population in the nursing profession in Israel. This phenomenon derives from the recent changes and needs of the UO society [36,37]. Women are the main providers in UO households because men devote their lives to religious study [38,39]. These women usually get married young and lack professional qualifications, which results in low-paying jobs. Therefore, for them, nursing studies offer an opportunity to acquire guaranteed employment and a stable source of income [40].

Additionally, there is growing awareness that community clinics and hospital wards must properly meet UO unique needs to maximize cultural competence and gender access [41,42,43]. This need was also recognized by the government, especially during the COVID-19 pandemic [44], and, therefore, there is a need for both male and female ultra-Orthodox nurses. As part of the national plan for integrating the UO population in academic nursing studies, several schools in Israel provide dedicated, gender-segregated programs to UO men [36] and women [37].

Nevertheless, nursing is not yet an acceptable profession in this community because it challenges a few of their religious norms, and the entry of UO men and women into the nursing profession entails multiple challenges [36,37]: (1) This community generally opposes integrating in academic studies; (2) Nursing is a science-based discipline, and potential students need a strong foundation of science. The elementary and high school programs of the UO education system lack knowledge and skills in many domains; and (3) The *Halakhah* (Jewish law) specifies strict sex-based roles to avoid physical or other connection between men and women [45].

Our study advances the understanding of the experiences of nurses from ethnic minority populations who serve the majority population, and, at the same time, could take advantage of their profession to help their minority community [20]. Research questions include:

1. What are the challenges of UO female nursing students in encounters with patients and health staff who belong to other communities?
2. How were these challenges manifested during the COVID-19 pandemic?
3. What are the challenges of UO female nursing students in their encounter with their community and how did these challenges manifest during the COVID-19 pandemic?

## 4. Materials and Methods

### 4.1. Study Settings

All participants were female nursing students at the Jerusalem College of Technology (JCT) [46]. JCT specializes in providing science and technology education to the religious Jewish community and about 45% of JCT’s 4700 students are UO Jews; among them, 55% are women. Nursing is the largest department of the institute, and out of the nursing students, 88% were women. Men and women study in separate campuses according to their religious norms. All research participants studied a unit in cultural competence as part of a mandatory first-year academic course named “Sociology of Health and Illness”. The course was monitored by the last author; classes were supplemented by theoretical reading and class discussions.

### 4.2. Study Design

Our main research tools included a questionnaire and focus-group interviews according to sequential explanatory mixed methods [47]. According to this strategy, the analysis of quantitative findings was followed by the analysis of focus-group interviews to explain the quantitative findings.

### 4.3. Data Collection

#### 4.3.1. Quantitative Phase

For the quantitative phase, we composed a questionnaire comprising 14 items ranked on a 1–10 Likert-scale, (ranging from “strongly disagree” to “strongly agree”). We adopted six items from the Hebrew version of the RCTSH Cultural Competence Assessment Tool (CCATool), student nurse specific, which consists of four sections measuring cultural awareness, cultural knowledge, cultural sensitivity, and cultural practice [48]. The questionnaire focused on the importance accorded by the students to the need of supplying medical information and guidelines with sensitivity to the beliefs, values, and norms of patients of different cultures, specifically in light of the experience during the COVID-19 pandemic. The questionnaire included items to assess the insights of the UO students, who constitute a large proportion of students at the college. Of the 235 female respondents, 111 (47%) were UO.

The questionnaire was administered to nursing students at JCT in June 2020, and 285 of them completed the questionnaire. We statistically analyzed the responses of 235 female students who completed the questionnaire. Fifty questionnaires were omitted from the study because they had a high percentage of missing data.

#### 4.3.2. Qualitative Phase

Six focus group were conducted between January and February 2021, with two to three students in each group (*n* = 15). The participants were all UO female nursing students from all four years of the nursing program at JCT. Each group contained students from the same school year. Purposive sampling was performed and participants were recruited by the first author who knew the participants well and thought that they would contribute to the research. Six students refused to participate in the study.

Each focus group interview lasted 60–90 min, conducted using a script designed under the guidance of the last author, a qualitative research expert. Following our quantitative phase results, the questions were specifically related to participants’ dilemmas around the decision to study nursing and their community’s response to that decision, as well as students’ experiences of encountering different cultures during their clinical rotations, most of which occurred during the COVID-19 pandemic. The first group was a pilot with three students from the second, third, and fourth years; their insights helped finalize the script questions.

All focus group were conducted online (via Zoom) and recorded with the participants’ consent. Audio recordings were later fully transcribed by a professional transcriptionist. Transcripts were compared to audiotapes to ensure accuracy by the last author.

### 4.4. Data Analysis

#### 4.4.1. Quantitative Phase

We conducted factor analysis using a varimax technique to define the categories within the questionnaire items. Twelve of the original fourteen items were categorized into four factors that were significant to the research questions.

Confidence in coping with the effects of racism at work as a nurse; sense of mission as a nursing student in using knowledge and authority in their community; cultural identity and community belonging; and cultural sensitivity. Cronbach’s α values for each category showed good internal consistency. Table 1 presents the items of each category, the number of items for each factor, and Cronbach’s α.

#### 4.4.2. Qualitative Phase

We chose an inductive approach, which means that the themes identified were closely linked to the data without trying to fit them into an earlier analytic theory [49]. We utilized thematic analysis—a recommended technique for analyzing focus group transcripts [50]. The six phases of thematic analysis as described by Braun and Clarke (2006) [51] were followed: (1) familiarizing with the data; (2) generating initial codes; (3) searching for themes; (4) reviewing themes; (5) defining and naming themes; and (6) producing the report. Phases one and two were conducted individually by the first and the last authors. The initial codes were compared at joint meetings. After comparing the two lists and reaching an agreement, a joint list was prepared. The third and fourth phases were jointly conducted by the two authors and shared with team members for feedback. The fifth and sixth phases were performed by the last author. The analysis was performed manually without the use of any software [52].

### 4.5. Trustworthiness

To establish the trustworthiness of the present study, we implemented various strategies. *Consistency* was achieved with all focus group sessions being conducted by a single researcher with a single set of questions during a short period [53]. *Credibility* [54] was achieved through practices, including: (1) *a thick description*: we provided in-depth illustrations and abundant details about the culture and social context of our study; (2) *triangulation*: we used multiple coding. Two researchers analyzed the data independently and compared their findings [55]; and (3) *multivocality*: we provided empathic understanding and space for a variety of opinions. To facilitate *transferability* [53], we reported in the article clear descriptions of the research context, selection procedure, characteristics of participants, data collection, and the process of analysis.

## 5. Results

### 5.1. Quantitative Phase

A series of independent *t*-tests were conducted to examine any statistically significant differences between the UO students and the general students in the factors identified in the questionnaire. Table 2 presents the results of independent *t*-tests.

As presented in Table 2, there is a statistically significant difference between the two groups in terms of the *sense of mission as a nursing student in using knowledge and authority in their community* (*p* = 0.01; t (233) = 2.46). The UO nursing students revealed a higher sense of mission toward their community (M = 6.67, SD = 2.46), compared to other nursing students. Differences related to other factors were not statistically significant between the groups.

Additionally, we conducted the Spearman’s correlation coefficient test to examine correlations between the various factors regarding the school year of the female students in the nursing study program. The results are shown in Table 3.

As presented in Table 3, there is a significant positive correlation of the school year with the *sense of mission as a nursing student in using knowledge and authority in their community* factor (*p* < 0.01; r = 0.28). This correlation signifies that as the student advances with nursing studies, the sense of mission toward her community increases. Additionally, there were significant positive correlations between the *sense of mission as a nursing student in using knowledge and authority in their community* factor and the *confidence to cope with the effects of racism at work as a nurse* factor (*p* < 0.05; r = 0.13); the *cultural identity and community belonging* factor (*p* < 0.05; r = 0.27); and the *cultural sensitivity* factor (*p* < 0.05; r = 0.14). Moreover, there was a significant positive correlation between the *confidence in coping with the effects of racism at work as a nurse* factor and the *cultural sensitivity* factor (*p* < 0.05; r = 0.40).

#### Additional Results

We also conducted Levene’s test for equality of variances of each item of the questionnaire to identify any significant statistical differences in specific items between the UO and other students and the non-UO students. The results are presented in Table 4.

As presented in Table 4, statistically significant differences exist between the UO and the non-UO students in four items: *The pandemic has made me critical of other people’s attitudes towards my community* (*p* < 0.001); *I used my authority as a nursing student to encourage others in my community to obey the instructions of the Ministry of Health during the pandemic* (*p* < 0.01); *I used my knowledge as a nursing student to guide others in my community on how to behave properly during the pandemic* (*p* < 0.01); and *I am very confident to challenge racism and discrimination toward clients* (*p* < 0.05).

### 5.2. Qualitative Phase

Two themes were identified in the analysis as follows (see Table 5 for quotations):

#### 5.2.1. Challenges in the Encounters Ultra-Orthodox Nursing Students Experience within Their Community

*Students had to overcome objections within their community in relation to exercising the students’ wish to enter nursing school.*

Most of the participants shared that they faced objections to academic studies from within their close community (high school teachers, friends, cousins, etc.). Some of them specifically faced objections against entering the nursing field and had to keep their plans secret until they began their studies. They all asked for permission from their parents and their rabbi prior to enrolling in nursing school. When asked, participants strongly recommended that other girls from their community study nursing. However, they did not see themselves as activists for social change for girls or women in their community. Rather they emphasized their personal interest in nursing and the mission they see in the nursing profession.

*Students expressed ambivalence about their role as health information providers to their community.*

Although they encountered objection, participants said that they are highly valued in their community for having studied nursing. Participants noted that people considered them as health authorities and sought their advice regarding health, even though they were still students. Participants explained they were flattered as it raised their confidence and self-pride. They felt obliged to share the health and medical information they acquired in the nursing school with their community. Nevertheless, participants expressed ambivalence since they hesitated to answer medical questions because, as they said, they are still students and preferred to direct such questions to a professional, such as a nurse or physician.

*Students described having mixed feelings in relation to providing care to patients from their community.*

Participants emphasized their mission as UO nurses familiar with the cultural sensibilities of their community. Participants mention their responsibility to help patients from their community feel comfortable when they arrive at general hospitals and clinics. However, a few of the participants noted that the cultural background they share with patients from their community was not always beneficial. Under certain circumstances, clinical procedures may create situations that conflict with cultural norms and embarrass both parties. For example, in cases where they needed to touch or even talk to young UO men, where both parties were aware of the restrictions about relations between men and women in UO communities. The participants explain how they overcome these difficulties and emphasized that in moments of embarrassment, they focus on their task and remind themselves that everything they are doing is professional with the purpose of helping the patient.

*Students mediated reliable health and health behavior information to their community at the time of the COVID-19 pandemic.*

The pandemic increased the participants’ sense of responsibility for conveying medical information to their community. They described their role during the COVID-19 pandemic amid massive confusion among their community members who have less exposure to smartphones, the Internet, television, and radio. Community members therefore sought and relied on COVID-19-related information from participants. Participants explained that they were surprised to see that the guidelines of the Ministry of Health were often misleading and sometimes contradictory of UO community customs. For example, during the holidays that occurred at the beginning of the epidemic, the directives of the Ministry of Health were to maintain social isolation and the UO people wanted to observe their custom of offering prayers in public. Participants described mediating for their close community between the formal guidelines of the Ministry of Health and those given by community leaders.

#### 5.2.2. Challenges in the Encounters of Ultra-Orthodox Nursing Students with Other Sections of Israeli Society

*Students appreciated the opportunity to challenge their perceptions about others outside of their community but also mentioned the difficulties involved.*

The experiences of nursing students during their clinical training provided them with the opportunity, sometimes the first opportunity they had experienced, to meet people from other sections of Israeli society, such as nonreligious Jews and non-Jews (i.e., patients from the Arab sector). Participants discussed how this experience was sometimes surprising and made them more open-minded although the encounters were challenging because of exposure to different—sometimes contradictory—values, norms, and customs. Few participants described feeling more comfortable with people from their own community—who they treated more warmly—than other patients, who they treated “by the book”.

*Students encountered racism during the COVID-19 pandemic, although they raised criticism towards the behavior of the members of their community.*

Interactions with patients and staff from various communities of the Israeli society exposed the participants to prejudices toward their community. Participants said that they learned for the first time that the public blames the UO community for the spread of the virus because they did not maintain social distance in the first period of the of the COVID-19 pandemic. The participants said that they are not always comfortable when others see them as representatives of the ultra-Orthodox society. For some participants, the exposure to stigmas about their community increased their sense of identification with their community. These participants were angry with the critical attitude of outsiders, since, as they said, the guidelines did not always reach the ultra-Orthodox community. Some saw their role as nurses as an opportunity to change these prejudices against their community. However, a few criticized the behavior of their community members during the pandemic.

## 6. Discussion

Previous studies have addressed the factors related to multicultural nurses’ experiences in encounters with the general population, as well as factors related to their work with patients from minority groups, especially from their community [14,15,17,18,19]. This study focused on female UO nursing students, their challenges, and dilemmas in their encounters with the general Israeli population, as well as their own community members, especially during the COVID-19 pandemic.

The nursing profession is noble, as nurses save human lives—a value highly regarded in the Jewish tradition and especially in the UO community [56]. However, the UO female nursing students are exceptional in their community that rejects academic pursuits. Their vocational nursing training in school, and later their profession, require extensive encounters with different worldviews, which can lead to a clash with their lifestyles [36].

*Ultra-Orthodox nursing students’ sense of mission*

The participants sense of mission was the main finding in both the quantitative and qualitative analyses in our study. Arieli and Hirschfeld (2010) [57] found that, compared to Palestinian–Israeli nursing students, most Jewish students’ reason for choosing nursing was an inner desire to serve. Pragmatic considerations for choosing nursing were mentioned but were usually presented by Jewish students as a relatively minor motivation. The quantitative phase of our study showed how the UO nursing students revealed a significantly higher sense of mission toward their community compared to the other nursing students in our study [t (233) = 2.46, 95% confident interval of the difference 0.15–1.39]. In the focus groups, participants emphasized nursing as a mission to help and serve others. They described their mission to provide reliable health-related information to their community. This was especially noticeable during the COVID-19 pandemic, which exposed the trust deficit between their community and health authorities [27].

Entering nursing school gives UO students an opportunity for social mobility [58]. They acquired higher education, and it will help them find a stable and well-paying job in the future, which will improve their social status [59,60]. Nursing education also gives participants in our study a chance for social mobility within their community because they hold valuable social capital: their professional—and potentially lifesaving—knowledge about health issues [14,15,19]. However, although these students are pioneers of their community in terms of entering nursing school, the participants in the focus groups emphasized not regarding themselves as “flag bearers” of social change for other women in their community. They stressed that they entered nursing school after receiving approval from the rabbinical authority and their parents. They reported remaining committed to the values of their community and stated that their choice of the nursing profession was not driven by a desire for social mobility. They were happy to recommend this direction to other UO girls.

*Ultra-Orthodox nursing students’ culture competence challenges*

Our study shows the dilemmas and challenges that the UO nursing students faced in their intercultural encounters with the general society, coming—as they did—from a closed, distinct group that faithfully upholds religious and traditional values and practices [61]. At the same time, they discussed their struggle when facing patients from their own community. Their deep acquaintance with the cultural norms of the UO community brings the UO participants to tread cautiously so as not to offend these patients.

The findings of studies on changes in cultural competence over the years in nursing schools are contradictory. A few studies indicate that there has been no change over the years [62,63], whereas others show that there has been a change [64]. The cultural competence factors examined in our study sample, especially the sense of mission, increased with advances in nursing studies. This was true for the UO participants, as well as the non-UO participants. It is in line with the significant positive correlations between the examined factors, among themselves, and in relation to the school year of the program studies, as it is well confirmed by Spearman’s correlation coefficient test.

Utilizing the CCATool [48], Repo et al. (2017) [4] found cultural competence to be positively associated with Finnish nursing students’ minority backgrounds and frequency of interacting with different cultures. Following the above finding, we assumed that in our study, as students’ progress academically, they perform more practical training in hospitals and in the community and interact with a diverse population both among patients and staff, which enhances their sensitivity toward other cultures. The academic progress of our UO participants also enhances their sense of responsibility toward their community.

Repo et al. (2017) [4] also reported that students with a minority background had higher cultural competence than general Finnish students. This has also been reported by reported by Mesler (2014) [65]. We agree with Repo et al.’s ([4], p. 102) explanation that “this may be due to the fact that minority background students already are culturally aware since they have had to reflect on their own ethnic and cultural identity while living as foreigners in Finland.” In our focus groups, the UO participants declared that they believe in equal medical care for all people and that they follow this tenet at work. Compared to other students in our sample, the UO participants were not only more sensitive to the issue of racism toward patients, but they felt more confident in dealing with it. It was manifested in a statistically significant difference between the UO and the non-UO students in the quantitative phase in the item: *I am very confident to challenge racism and discrimination toward clients*. However, they did not state that they were trying to develop awareness or acquire knowledge about patients from other communities.

*Ultra-Orthodox nursing students in the COVID-19 pandemic*

Their clinical experience, especially when the COVID-19 pandemic first appeared, exposed our study participants to the ignorance of parts of their community members about health issues, their lack of access to the latest and most reliable information due to cultural–religious restrictions, and the serious consequences that all of these had on the community members’ health.

Previous studies [14,15,17,18] have noted language and communication problems as barriers, especially for minorities, in gaining access to health services. The UO Jews constitute a minority group in Israeli society, although most of them speak Hebrew, which is the common language of Israel [66]. However, UO communities lack health literacy [27], which refers to “the degree to which individuals have the ability to find, understand, and use information and services to inform health-related decisions and actions for themselves and others” [67].

The participants in the focus groups shared that their community members recognized them as a source of reliable medical information, and they often made the guidelines of the Ministry of Health accessible to their community. It is in line with the findings of the quantitative phase, which showed significant differences between the UO and the non-UO students in the items: *I used my authority as a nursing student to encourage others in my community to obey the instructions of the Ministry of Health during the pandemic; I used my knowledge as a nursing student to guide others in my community on how to behave properly during the pandemic*.

The COVID-19 period accentuated for the participants not only their roles within their community, but also what the public thought about their community. The UO community faced instances of racism during the COVID-19 pandemic. There were cases where people blamed them for spreading the disease by disobeying the official instructions for limiting the spread of the virus [27].

Some of the UO participants in our research were exposed, due to their work as nurses, to the prejudices about their community for the first time, largely because the UO community stays isolated from the general society as part of their religious ideology. The UO students described how patients regarded them as representatives of the UO society, which was not always appropriate, especially when their community was blamed for the spread of the disease. It is in line with the statistically significant difference between the UO and the non-UO students in the quantitative phase in the item: *The pandemic has made me critical of other people’s attitudes towards my community.* Moreover, a few participants in the focus groups recounted the comments of the medical staff, especially nurses who worked with them. Iheduru-Anderson et al. (2021) [68] (pp. 122–123) found that “racism in nursing remains under-investigated. There is a collective denial and a culture of silence around racism, which perpetuate systemic racism and its consequences on the nursing profession…” Following their experience, the UO participants in our focus groups acknowledged the importance of treating members of their own community with greater warmth because of the difficulties and barriers they face in the society.

### Limitations

This study has several limitations. First, the small number of items in the questionnaire limits the number of identified factors in the factor analysis. Hence, significant differences between the UO and the non-UO students in specific items, which are relevant to the research questions, are found in Levene’s test for equality of variances of each item but are not revealed in the comparison of the factors between the two groups. Using a larger number of items might define factors, which would include the “missed” items within the categorized groups of items. However, the factors are highly supported by the themes identified in the analysis of the qualitative phase of this study. Second, in the quantitative phase, we compared the UO students—this study’s focus—with the non-UO students in the same college. However, the latter are also religious Jews and do not represent the secular section of Israeli society. This might limit the generalization of the study. Our assumption is that because the non-UO participants in the study resemble general Israeli women in terms of their previous education and other social variables, such as participating in the military service (or national service instead) [69], they are integrated into Israeli society, and, therefore, can represent a control group for the UO participants.

## 7. Conclusions

Our research shows that transcultural nursing students play a decisive role in mediating updated and accurate health information to their community, especially at times of crises, from the initial stages of their training. However, they face dilemmas both inside and outside their respective communities. We believe that insights derived from this study can be utilized to build programs to address the challenges of nurses from minority communities and transcultural nurses. At the policy level, we join the call of Drach-Zahavy et al. (2021) [70] to prepare in advance a crisis plan for nursing schools that could be rapidly implemented, and to incorporate in the nursing curricula crisis-related topics, such as developing flexible coping strategies. We propose that these policies should leverage the substantial potential of transcultural nursing students in working with their communities and consider these students’ distinct needs and dilemmas. This will help channelize the social role of transcultural nurses for conveying otherwise inaccessible information to isolated communities.

## Figures and Tables

**Table 1:** Categories, Items, and Cronbach’s α.

Category	Item Number		Number of Items	Cronbach’s α
*Confidence in coping with the effects of racism at work as a nurse*	13	I am able to incorporate clients’ cultural beliefs into the care and treatment I provide	4	0.75
	14	I am very confident to challenge racism and discrimination toward clients		
	15	I am very confident to challenge racism and discrimination toward staff		
	16	I am very confident to challenge racism and discrimination toward me		
*Sense of mission as a nursing student in using knowledge and authority in their community*	5	The pandemic strengthened my sense of mission and choice in the nursing profession	3	0.75
	7	I used my authority as a nursing student to encourage others in my community to obey the instructions of the Ministry of Health during the pandemic		
	8	I used my knowledge as a nursing student to guide others in my community on how to behave properly during the pandemic		
*Cultural identity and community* *belonging*	2	The pandemic taught me that there are differences between my cultural identity and other groups in Israeli society	3	0.65
	3	The pandemic has made me critical of the behavior of members of my community		
	4	The pandemic has made me critical of other people’s attitudes towards my community		
*Cultural Sensitivity*	9	I am very comfortable working with people whose beliefs, values, and practices are different from my own	2	0.61
	10	I am very confident of my ability to establish trust and show respect and empathy to people from other cultures		

**Table 2:** Independent Samples *t*-Test (Factors).

Category	Ultra-Orthodox *n* = 111		Non-Ultra-Orthodox *n* = 124		*t*-Value
	Mean (M)	Std. Deviation (SD)	Mean (M)	Std. Deviation (SD)	
*Confidence in coping with the effects of racism at work as a nurse*	6.82	1.81	6.47	1.59	1.66
*Sense of mission as a nursing student in using knowledge and authority in their community*	6.67	2.46	5.93	2.38	2.46 *
*Cultural identity and community belonging*	6.22	2.31	5.84	2.11	1.58
*Cultural sensitivity*	7.74	1.76	7.6	1.56	0.68

* *p* < 0.05.

**Table 3:** Spearman’s correlation coefficient between factors according to the school year of the studies.

	School Year	*Confidence in Coping with the Effects of Racism at Work as a Nurse*	*Sense of Mission as a Nursing Student in Using Knowledge and Authority in the Community*	*Cultural Identity and Community* *Belonging*	*Cultural Sensitivity*
**School Year**	1	0.02	0.28 **	0.03	−0.01
***Confidence in coping with the effects of racism at work as a nurse***		1	0.13 *	0.1	0.40 **
***Sense of mission as a nursing student in using knowledge and authority in their community***			1	0.27 **	0.14 *
***Cultural identity and community*** ***belonging***				1	0.06
***Cultural sensitivity***					1

* *p* < 0.05, ** *p* < 0.01.

**Table 4:** Independent Samples Test (Items).

Items		Students		
		Ultra-Orthodox	Non-Ultra-Orthodox	
		*n* = 111	*n* = 124	
No.	Item	Mean		Sig. (2-Tailed)
2	The pandemic taught me that there are differences between my cultural identity and other groups in Israeli society	6.370	6.310	0.859
3	The pandemic has made me critical of the behavior of members of my community	5.950	6.410	0.243
4	The pandemic has made me critical of other people’s attitudes towards my community	6.500	4.730	0.000 **
5	The pandemic strengthened my sense of mission and choice in the nursing profession	7.970	7.870	0.233
6	The pandemic raised concerns regarding my exposure to risks, when I become a nurse	4.960	5.580	0.105
7	I used my authority as a nursing student to encourage others in my community to obey the instructions of the Ministry of Health during the pandemic	6.250	5.080	0.004 **
8	I used my knowledge as a nursing student to guide others in my community on how to behave properly during the pandemic	6.000	4.900	0.006 **
9	I am very comfortable working with people whose beliefs, values, and practice are different from my own	7.02	6.96	0.821
10	I am very confident of my ability to establish trust and show respect and empathy to people from other cultures	8.56	8.3	0.170
11	I am aware of my ethnic and cultural identities	8.710	9.000	0.202
12	I am updated regarding the culture and social status of my clients	7.2	7.11	0.710
13	I am able to incorporate clients’ cultural beliefs into the care and treatment I provide	6.65	6.25	0.134
14	I am very confident to challenge racism and discrimination toward clients	7.53	6.93	0.029 *
15	I am very confident to challenge racism and discrimination toward staff	6.77	6.51	0.398
16	I am very confident to challenge racism and discrimination toward me	6.530	6.220	0.358

* *p*-value < 0.05; ** *p*-value < 0.01.

**Table 5:** Qualitative results—Selected quotes.

Theme Number	Selected Quotes
**Theme 1:** Challenges in the encounters Ultra-Orthodox nursing students experience within their community	*Students had to overcome objections within their community in relation to exercising the students’ wish to enter nursing school.* “I know this is not an acceptable step and many look at it as ‘What are you doing? Are you going to college? To be a nurse?’ But I try not to look at what society now tells me. I went to my rabbi and consulted with him and my parents. Then I decided—I go for it. Of course, I will not do anything that is unacceptable” (C2) “… I do not see myself as a pioneer… that’s what was right for me… This profession is a mission. It gives satisfaction…” (C2) “I felt I had enough resilience to go and do it [study nursing]. That does not mean I would recommend it to anyone else. Each one should think with herself and with her rabbis.” (C1) “My high school principal told me I was an educational failure for him… and that it [nursing] was a bad profession to study, that the learning topics are inappropriate and ‘God forbid.’ … it was hard, but I had all the support I needed. It was something I wanted, and no one could move me, so I said to myself ‘O.k. we do not have the same worldview’ and that’s it.” (P1) *Students expressed ambivalence about their role as health information providers to their community.* “… I also have heard of breast cancer. I called my grandmother and started to publicize it. ‘Get examined as soon as you can and do it frequently.’ … I also think that in the future … the more I am exposed to [medical] cases, and I know what happens, I will warn more people. I don’t think I will advise them medically, but I will make recommendations.” (B5) “… Most of the people who asked questions and consulted with me were very UO. They had been turning to me for advice since they heard I had started to study nursing. Even in the first year, when you start to study anatomy, they are convinced you are already a doctor.” (D5) “I don’t often take the responsibility, but sometimes it is my cousins or other family members or neighbors—people close to me, so I can tell them what I think they should do and whom to consult. They should turn to someone who knows, someone who is qualified.” (D5) “We [nursing students] know where and whom to turn to: the Internet, a physician, or a nurse; people constantly ask me.” (C5) “… my brother says, ‘I am asking you because … I don’t know the most reliable source, and you know exactly where to look.’” (C4) “Yes, many people turned to me, and it is extremely flattering. I also feel that I know something—maybe not that much, but more than the average person.” (B1) “… It’s really amazing when people see you as someone special, not just a child.” (B3) *Students described having mixed feelings in relation to providing care to patients from their community.* “I see the possibility of adapting medical care to the needs of the community. I see this as one of my goals as a nurse and as an UO Jew.” (B1) “… Patients from the UO community understand the nuances. They identify me as one of their own, so we speak differently to each other, and they are happy because I understand them. It is a fact that we need more ultra-Orthodox nurses in the field.” (C1) “When they [UO patients] see me taking care of them, they feel more comfortable. I am dressed the same way they are, and I speak their language and feel that they feel more comfortable to approach me. I sit at the nursing station and when they arrive, they turn to me before turning to anyone else.” (C5) “… You really see that UO people are happy to see that there is someone from their community in the field [of nursing]… It’s nice… I’m also happy to meet patients like me… the whole ‘cultural competence’ issue… if you do not have to do special steps to adapt yourself [to the patient] it is much easier.” (P1) “… Every time an UO patient arrives, especially in my department, it is really difficult to take care of them … let’s say, it is a 20-year-old boy who studies in a yeshiva—I know exactly where he is coming from and that I am the only woman who has ever touched him outside of his family (she grins). Just because I know where he is coming from, it is not so pleasant for me.” (C3) “… As women in the ultra-Orthodox community, we do not touch men… It is because of ‘halakha’ roles…. This is how we grew up and this is how we were educated… I will feel uncomfortable to insert a catheter to a man… maybe I will choose a women’s ward or an emergency room where there is less need to take care of intimate parts of the body” (B1) *Students mediated reliable health and health behavior information to their community at the time of the COVID-19 pandemic.* “You have to know how to approach them [the UO community]. You can’t tell them on television … because they don’t watch television. However, as soon as their rabbi tells them to do so, there is a lockdown—then they stay indoors. You have to reach people in a way they prefer.” (C1) “My mother’s friends call me regarding COVID-19; not that I know what to answer, but they ask. Let’s say, they want me to arrange a COVID-19 test for them: when and where they should do it. It is strange that although I am much younger to them, they call and ask.” (B3) “The information that reaches the UO community is sometimes confusing, and they don’t understand it. Neither do secular people understand these things scientifically. In such situations, I can be a representative and can transmit information.” (C2) “More than asking about what happens inside, they [the UO people] ask what happens outside because they don’t know what is going on. They lack information … reliable information. They don’t know what is happening outside of their world, if they are missing information. I am a reliable source to them regarding COVID-19.” (C2) “I don’t feel like I have a mission, but I can make people more aware that if they think they have nothing to be afraid of COVID-19, there is still something to be afraid of.” (D2) “Our rabbi said, if someone in the family is sick with COVID-19, the entire family must be in lockdown. You [as a nurse] have to relate to this source of authority and not only to what the Ministry of Health says. It’s complicated. All of the responsibility is on you.” (B3)
**Theme 2:** Challenges in the encounters of Ultra-Orthodox nursing students with other sections of Israeli society	*Students appreciated the opportunity to challenge their perceptions about others outside of their community but also mentioned the difficulties involved.* “When you know team members from other communities then you own, your viewpoint changes. We all have prejudices about other societies. If we do not exercise some thinking before, we may judge people automatically by how they look to us. Once you get to know a person a little deeper, stigmas come down” (D1). Now I’m in a clinic where 90% of the patients are Arabs. Really until now, I did not think that I would have to think about their language and culture… it is important to recognize it as a nurse, …” (B3) “In high school, you are protected. Those who teach you—these are people from your community. You study with people from your community. Suddenly you come to the academy and the lecturer is, let’s say, is a very smart, valued woman, that has a different way of life from you… It could raise thoughts… You suddenly meet the world, and it looks a bit different… very different then what you were told you” (C1) “I had stigmas but when I did my clinical practice, I saw that… there are no such stigmas. I treat a person, a patient, not his race, his religion. Those stigmas just went down, and I saw it was not an issue” (B5) “This [nursing] also connects me to the rest of the world; the feeling that we are together, united, closer, the minute that I can help them [nonreligious jews]. I think that from this perspective, it is possible that I will succeed in changing the way people look at my community.” (B5) “… telling you how to treat an Arab versus practically take care of an Arab patient is very different. It sounded much harder to me then my experience when it actually happened. I found myself smiling at them, asking ‘How are you?’ and treat them as human beings.” (C1) “I saw this during the COVID-19 pandemic … patients from other cultural background arrived and they had different opinions than mine. I must know how not to oppose them. I have to treat them in such a way that they accept me taking care of them, regardless of my own UO background.” (B3) “When I treat UO patients I feel … good that I am taking care of them. Now, my work is easier because I know how to relate to UO women … Arab women—I can only say the few words in Arabic; secular women—it’s not that I don’t show empathy or concern, but it’s just easier for me with UO women as someone who comes from this same background. I think most people are like this. If an Arab is treating an Arab, it will be easier for them than treating a Jew.” (C2) *Students encountered racism during the COVID-19 pandemic although they raised criticism towards the behavior of the members of their community.* “There is a remark that I as an UO often hear: ‘O.K. You are not like everyone else. You are not the standard UO woman, so it’s ok.’ They often get me annoyed because none of the UO are ‘standard’ UO. So, I wonder, what kind of UO people are you talking about?” (B1) “The entire period of the COVID-19 pandemic … changed what we [UO] thought about the general public because we felt there was immense antagonism against us. Not just against the extremists, but against ordinary people also. It was unfortunate that relations that had developed over years, were upended in one year.” (B1) “There is a stigma of mass infection in our community [i.e., UO neighborhoods]; that we live in the most contagious part of the city, and that no one is obeying the lockdown.” (B1) “I live in Bnai Brak [the largest UO city in Israel] and I hear a lot of stories about us. I really don’t like these stories. So, I feel that I am more critical [toward my community] than others who criticize me as part of this community.” (C3) “… This is something that really bothers me about the UO community: a kind of blocking things out, brainwashing without using their own mind to think. This really gets me angry. Now [during the pandemic] it emerges in more ways.” (D1)

## Data Availability

The study did not report any data (all data is reported in the article).

## References

[1] Tosun B., BENEFITS Group (2021). Addressing the effects of transcultural nursing education on nursing students’ cultural competence: A systematic review. Nurse Educ. Pract..

[2] Calvillo E., Clark L., Ballantyne J.E., Pacquiao D., Purnell L.D., Villarruel A.M. (2009). Cultural competency in baccalaureate nursing education. J. Transcult. Nurs..

[3] Campinha-Bacote J. (2011). Delivering patient-centered care in the midst of a cultural conflict: The role of cultural competence. Online J. Issues Nurs..

[4] Repo H., Vahlberg T., Salminen L., Papadopoulos I., Leino-Kilpi H. (2017). The cultural competence of graduating nursing students. J. Transcult. Nurs..

[5] Schuster M., Elroy I., Elmakais I. (2017). We are lost: Measuring the accessibility of signage in public general hospitals. Lang. Policy.

[6] Schuster M., Elroy I., Rosen B. (2018). How culturally competent are hospitals in Israel?. Isr. J. Health Policy Res..

[7] Govere L., Govere E.M. (2016). How effective is cultural competence training of healthcare providers on improving patient satisfaction of minority groups? A systematic review of literature. Worldviews Evid.-Based Nurs..

[8] Squires A. (2017). Evidence-based approaches to breaking down language barriers. Nursing.

[9] Halbert C.H., Armstrong K., Gandy O.H., Shaker L. (2006). Racial differences in trust in health care providers. Arch. Intern. Med..

[10] Ume-Nwagbo P.N. (2012). Implications of nursing faculties’ cultural competence. J. Nurs. Educ..

[11] Gaston-Johansson F., Hill-Briggs F., Oguntomilade L., Bradley V., Mason P. (2007). Patient perspectives on disparities in healthcare from African-American, Asian, Hispanic, and Native American samples including a secondary analysis of the Institute of Medicine focus group data. J. Natl. Black Nurses Assoc..

[12] Meghani S.H., Brooks J.M., Gipson-Jones T., Waite R., Whitfi eld-Harris L., Deatrick J.A. (2009). Patient-provider race-concordance: Does it matter in improving minority patients’ health outcomes. Ethn. Health.

[13] Lo M.C.M., Bahar R. (2013). Resisting the colonization of the lifeworld? Immigrant patients’ experiences with co-ethnic healthcare workers. Soc. Sci. Med..

[14] Lo M.C.M., Nguyen E.T. (2018). Caring and carrying the cost: Bicultural Latina nurses’ challenges and strategies for working with coethnic patients. RSF Russell Sage Found. J. Soc. Sci..

[15] Lo M.C.M., Nguyen E.T. (2021). Resisting the racialization of medical deservingness: How Latinx nurses produce symbolic resources for Latinx immigrants in clinical encounters. Soc. Sci. Med..

[16] Seto A., Forth N.L.A. (2020). What is known about bilingual counseling? A systematic review of the literature. Prof. Couns..

[17] Dawson M.A. (2021). Black nurses collaborative approach to addressing COVID-19 in Black communities. J. Racial Ethn. Health Disparities.

[18] Delgdo-Romero E.A., De Los Santos J., Raman V.S., Merrifield J.N., Vazquez M.S., Monroig M.M., Bautista E.C., Duran M.Y. (2018). Caught in the middle: Spanish-speaking bilingual mental health counselors as language brokers. J. Ment. Health Couns..

[19] Keshet Y., Popper-Giveon A. (2016). Work experiences of ethnic minority nurses: A qualitative study. Isr. J. Health Policy Res..

[20] Cuellar N.G., Aquino E., Dawson M.A., Garcia-Dia M.J., Im E.O., Jurado L.F.M., Lee Y.S., Littlejohn S., Tom-Orme L., Toney D.A. (2020). Culturally congruent health care of COVID-19 in minorities in the United States: A clinical practice paper from the National Coalition of Ethnic Minority Nurse Associations. J. Transcult. Nurs..

[21] Biale D., Assaf D., Brown B., Gellman U., Heilman S.C., Rosman M., Sagiv G., Wodzinsky M. (2018). Hasidism: A New History.

[22] Rubinstein A., Gartner L.P., Baskin J.R., Morris B.J., Jacobs L., Shatz-Uffenheimer R. (2007). Hasidism. The Encyclopaedia Judaica.

[23] Taragin-Zeller L., Rozenblum Y., Baram-Tsabari A. (2020). Public engagement with science among religious minorities: Lessons from COVID-19. Sci. Commun..

[24] Malach G., Cahaner L. (2020). Statistical Report on Ultra-Orthodox Society in Israel. The Israel Democracy Institute. https://www.idi.org.il/books/33367.

[25] Haredi Institute for Public Affairs (2020). The Coronavirus Crisis in the Ultra-Orthodox Population in Israel [Hebrew]. https://machon.org.il/wp-content/uploads/2020/06/d79ed7a9d791d7a8-d7a0d792d799d7a3-d794d7a7d795d7a8d795d7a0d794-d795d794d797d7a8d793d799d79d-d791d799d7a9d7a8d790d79c-d7a1d795d7a4d799.pdf.

[26] Israeli Central Bureau of Statistics (2018). Society in Israel, Report Number 10: Religion and Self-Definition of Level of Religiosity [Hebrew]. https://www.cbs.gov.il/he/publications/DocLib/2018/rep_10/h_print.pdf.

[27] Zalcberg S., Zalcberg Block S. (2021). COVID-19 amongst the ultra-Orthodox population in Israel: An inside look into the causes of the high morbidity rates. Contemp. Jew..

[28] Orr Z., Unger S., Finkelstein A. (2021). Localization of human rights of people with disabilities: The case of Jewish ultra-Orthodox people in Israel. Hum. Rights Q..

[29] Orr Z., Unger S., Finkelstein A. (2021). The challenges and dilemmas of local translators of human rights: The case of disability rights among Jewish ultra-Orthodox communities. J. Hum. Rights.

[30] Vardi M., Orr Z., Finkelstein A., Markovich D.Y., Golan D., Shalhoub-Kevorkian N. (2019). Civic engagement of students from minority groups: The case of ultra-Orthodox students and communities in Jerusalem. Understanding Campus-Community Partnerships in Conflict Zones.

[31] Golan A., Campbel H.A. (2015). Strategic management of religious websites: The case of Israel’s Orthodox communities. J. Comput.-Mediat. Commun..

[32] Gonen A., Sharon D., Lev-Ari L., Strauss E., Segev R. (2016). The impact of nursing students’ cultural diversity on the intention and attitudes toward the use of information technology. J. Transcult. Nurs..

[33] Raz A., Keshet Y., Popper-Giveon A., Karkabi M.S. (2021). One size does not fit all: Lessons from Israel’s COVID-19 vaccination drive and hesitancy. Vaccine.

[34] Orr Z., Erblich T., Unger S., Barnea O., Weinstein M., Agnon A. (2021). Earthquake preparedness among religious minority groups: The case of the Jewish ultra-Orthodox society in Israel. Nat. Hazards Earth Syst. Sci..

[35] Coleman-Brueckheimer K., Spitzer J., Koffman J. (2009). Involvement of Rabbinic and communal authorities in decision-making by haredi Jews in the UK with breast cancer: An interpretative phenomenological analysis. Soc. Sci. Med..

[36] Haron Y., Azuri P. (2016). Integrating ultra-Orthodox Jewish men in academic nursing training. J. Transcult. Nurs..

[37] Segev R., Strauss E. (2020). Forming an affiliation between two culturally different academic institutions of nursing studies. Open Nurs..

[38] Kolikant Y.B.-D., Genut S. (2017). The effect of prior education on students’ competency in digital logic: The case of ultraorthodox Jewish students. Comput. Sci. Educ..

[39] Buchbinder E., Sigad L., Strier R., Eisikovits Z. (2015). Working poor ultra-Orthodox Jewish women and men: Between economic distress and meaning based on faith. J. Poverty.

[40] Kulik L. (2016). Explaining employment hardiness among women in Israel’s ultraorthodox community: Facilitators and inhibitors. J. Career Assess..

[41] Gabbay E., McCarthy M.W., Fins J.J. (2017). The care of the ultra-orthodox Jewish patient. J. Relig. Health.

[42] Sharmila D. (2005). Mental health and religion in Israel’s ultra-Orthodox Jews. Lancet.

[43] Snyder J., Sommer B. (1995). Special considerations for Orthodox Jewish patients in the emergency departments. J. Emerg. Nurs..

[44] Waitzberg R., Davidovitch N., Leibner G., Penn N., Brammli-Greenberg S. (2020). Israel’s response to the COVID-19 pandemic: Tailoring measures for vulnerable cultural minority populations. Int. J. Equity Health.

[45] Foscarini G. (2014). Ultra-orthodox Jewish women go to work: Secular education and vocational training as sources of emancipation and modernization. Ann. Di Ca’ Foscari Ser. Orient..

[46] Finkelstein A., Orr Z. (2021). Does volunteering change attitudes towards people with disabilities? A qualitative study of the experience of orthodox Jewish nursing students. Nurse Educ. Pract..

[47] Shorten A., Smith J. (2017). Mixed methods research: Expanding the evidence base. Evid.-Based Nurs..

[48] Papadopoulos I., Tilki M., Ayling S. (2008). Cultural competence in action for CAMHS: Development of a cultural competence assessment tool and training programme. Contemp. Nurse.

[49] Patton M.Q. (1990). Qualitative Evaluation and Research Methods.

[50] Breen R.L. (2006). A practical guide to focus-group research. J. Geogr. High. Educ..

[51] Braun V., Clarke V. (2006). Using thematic analysis in psychology. Qual. Res. Psychol..

[52] Basit T. (2003). Manual or electronic? The role of coding in qualitative data analysis. Educ. Res..

[53] Graneheim U.H., Lundman B. (2004). Qualitative content analysis in nursing research: Concepts, procedures and measures to achieve trustworthiness. Nurse Educ. Today.

[54] Tracy S.J. (2010). Qualitative quality: Eight “big-tent” criteria for excellent qualitative research. Qual. Inq..

[55] Johnson R., Waterfield J. (2004). Making words count: The value of qualitative research. Physiother. Res. Int..

[56] Spiegel E. (2011). “Talmud Torah is Equivalent to All”: The Ultra-Orthodox (Haredi) Education System for Boys in Jerusalem.

[57] Arieli D., Hirschfeld M.J. (2010). Teaching nursing in a situation of conflict: Encounters between Palestinian-Israeli and Jewish-Israeli nursing students. Int. Nurs. Rev..

[58] Snee H., Goswami H. (2021). Who cares? Social mobility and the ‘class ceiling’ in nursing. Sociol. Res. Online.

[59] Brown P. (2013). Education, opportunity and the prospects for social mobility. Br. J. Sociol. Educ..

[60] Payne G. (2012). A new social mobility? The political redefinition of a sociological problem. Contemp. Soc. Sci..

[61] Kolikant Y.B.-D., Genut S. (2021). Change in Order not to Change: Ultraorthodox Hasidic Women’s Experience in Studying Computer Science. Comput. Sci. Educ..

[62] Rahma N.F., Novieastari E. (2019). Differences in cultural competence between nursing students in academic and professional programs. Enferm. Clin..

[63] Seidel Glass P.E. (2013). Differences among Undergraduate and Graduate Nursing Students’ Cultural Competency. Ph.D. Thesis.

[64] Witt A.J. (2016). The Impact of Cultural Encounters on the Cultural Competence of Baccalaureate Nursing Students. Ph.D. Thesis.

[65] Mesler D.M. (2014). A comparative study of cultural competence curricula in baccalaureate nursing programs. Nurse Educ..

[66] Keren-Kratz M. (2016). Westernization and Israelization within Israel’s extreme Orthodox Haredi society. Isr. Stud. Rev..

[67] (2021). Center for Disease Control and Prevention What Is Health Literacy?. https://www.cdc.gov/healthliteracy/learn/index.html.

[68] Iheduru-Anderson K., Shingles R.R., Akanegbu C. (2021). Discourse of race and racism in nursing: An integrative review of literature. Public Health Nurs..

[69] Genut S., Ori B., Ben-David Kolikant Y. (2019). Factors influencing women’s decision to study computer science: Is it context dependent?. Issues Inf. Sci. Inf. Technol..

[70] Drach-Zahavy A., Goldblatt H., Admi H., Blau A., Ohana I., Itzhaki M. (2022). A multi-level examination of nursing students’ resilience in the face of the COVID-19 outbreak: A cross-sectional design. J. Adv. Nurs..

